# Implementation of Genomic Prediction in *Lolium perenne* (L.) Breeding Populations

**DOI:** 10.3389/fpls.2016.00133

**Published:** 2016-02-12

**Authors:** Nastasiya F. Grinberg, Alan Lovatt, Matt Hegarty, Andi Lovatt, Kirsten P. Skøt, Rhys Kelly, Tina Blackmore, Danny Thorogood, Ross D. King, Ian Armstead, Wayne Powell, Leif Skøt

**Affiliations:** ^1^Manchester Institute of Biotechnology, University of ManchesterManchester, UK; ^2^Institute of Biological, Environmental and Rural Sciences, Aberystwyth UniversityAberystwyth, UK; ^3^CGIAR Consortium, CGIAR Consortium OfficeMontpellier, France

**Keywords:** perennial ryegrass, genomic selection, BLUP, machine learning, forage crop

## Abstract

Perennial ryegrass (*Lolium perenne* L.) is one of the most widely grown forage grasses in temperate agriculture. In order to maintain and increase its usage as forage in livestock agriculture, there is a continued need for improvement in biomass yield, quality, disease resistance, and seed yield. Genetic gain for traits such as biomass yield has been relatively modest. This has been attributed to its long breeding cycle, and the necessity to use population based breeding methods. Thanks to recent advances in genotyping techniques there is increasing interest in genomic selection from which genomically estimated breeding values are derived. In this paper we compare the classical RRBLUP model with state-of-the-art machine learning techniques that should yield themselves easily to use in GS and demonstrate their application to predicting quantitative traits in a breeding population of *L. perenne*. Prediction accuracies varied from 0 to 0.59 depending on trait, prediction model and composition of the training population. The BLUP model produced the highest prediction accuracies for most traits and training populations. Forage quality traits had the highest accuracies compared to yield related traits. There appeared to be no clear pattern to the effect of the training population composition on the prediction accuracies. The heritability of the forage quality traits was generally higher than for the yield related traits, and could partly explain the difference in accuracy. Some population structure was evident in the breeding populations, and probably contributed to the varying effects of training population on the predictions. The average linkage disequilibrium between adjacent markers ranged from 0.121 to 0.215. Higher marker density and larger training population closely related with the test population are likely to improve the prediction accuracy.

## Introduction

Genetic improvement of crops involves the selection of plants with superior characteristics in terms of traits that are considered important. This could be yield (biomass or seed), resistance to diseases and pests and better tolerance to abiotic stress. The selection criteria have been and still are based largely on phenotypic performance. Phenotypic assessment can be time consuming and laborious, particularly for perennial crops. There is pressure to increase agricultural output at a faster rate to keep up with population growth and reduced area available for agricultural production. Molecular marker assisted selection (MAS) represents a way of potentially reducing the time and effort needed for phenotypic testing (Lande and Thompson, 1990; Dekkers and Hospital, 2002; Xu and Crouch, 2008). The success of MAS is dependent upon sufficient linkage disequilibrium (LD) between a marker and the phenotypic QTL (quantitative trait locus), and the QTL explaining a substantial proportion of the variation for the trait. Often, this is not the case, and the association between marker and QTL is not significant, and thus discarded. Therefore, the use of MAS in plant breeding has not been widespread. Recent improvements in genotyping techniques have made it possible to cover the genome with densely populated molecular markers, and this has paved the way for genome wide association studies (GWASs) (Rafalski, 2002; Flint-Garcia et al., 2003) in which marker-trait associations can be identified in breeder relevant and more diverse populations, rather than bi-parental mapping populations. The disadvantages of this approach includes low statistical power from small population sizes, confounding population structure of the germplasm used, and overestimation of the effect of few significant marker associations with QTL (Heffner et al., 2009).

Genomic selection (GS) represents a way of dealing with many of the problems of current MAS methodology. The term was first used by Meuwissen et al. (2001) to describe the use of genome wide molecular markers to simultaneously estimate the effect of all markers across the genome, irrespective of whether they are significant, in order to calculate a genomically estimated breeding value (GEBV) of selection candidates. GS depends upon the establishment of a training population, for which both phenotypic and genotypic data are available. The marker effects calculated from these data can be used to estimate the breeding values in populations with only genotypic data available (Meuwissen et al., 2001; Heffner et al., 2009). In terms of prediction methods the most widely used are the genomic or ridge regression. BLUP (best linear unbiased prediction) and other penalized regression methods (Gianola et al., 2006; de los Campos et al., 2009; Li and Sillanpää, 2012) and various Bayesian techniques (Meuwissen et al., 2001; de los Campos et al., 2009; Habier et al., 2011). However these techniques do not explicitly account for interactions. There is currently considerable interest in applying machine learning (ML) to science, and reviews have recently appeared (Ghahramani, 2015; Jordan and Mitchell, 2015). These methods are increasingly being applied in GWASs and GS (Dudoit et al., 2002; Long et al., 2007; Ziegler et al., 2007; Szymczak et al., 2009; Ogutu et al., 2011, 2012; Ornella et al., 2014; Spindel et al., 2015). ML algorithms are well suited to application in plant-breeding datasets. Most are easy to use and are easily available in a variety of implementations. Many methods perform attribute selection (e.g., lasso, regression trees) or assign importance scores to variables (e.g., random forest, boosted trees). Some methods, such as tree based approaches, do not require any assumptions about the underlying trait (e.g., additivity of effects, the numbers and size of interactions, depth of interactions etc.) and are able to capture complex non-linear relationships between response and regressors.

Genomic selection is an attractive alternative to classic selection methods, and it has been adopted in animal breeding, particularly dairy cattle (Schaeffer, 2006; Pryce and Daetwyler, 2012; Hayes et al., 2013b). The uptake of GS has been slower in plant breeding, but is now gathering pace. Many papers have assessed the potential use of GS in simulation and empirical studies of some of the major crops (Bernardo and Yu, 2007; Heffner et al., 2009, 2010, 2011; Piepho, 2009; Zhong et al., 2009; Jannink et al., 2010; Albrecht et al., 2011; Poland et al., 2012; Zhao et al., 2012, 2013; Xu, 2013; Bentley et al., 2014; Jarquin et al., 2014; Wang et al., 2014; Xu et al., 2014). The applicability of GS in perennial crops such as trees and forages is even more appealing, due to the possibility of significantly reducing the length of the breeding cycle (Grattapaglia and Resende, 2011). Some empirical studies in trees suggest reasonable prediction accuracies can be obtained (Resende et al., 2012a,b; Zapata-Valenzuela et al., 2012; Beaulieu et al., 2014). Two factors need to be taken into consideration when dealing with breeding in many forage crops such as perennial ryegrass. Firstly, the performance of individual spaced plants generally does not correlate well with the phenotype in sward for many economically important traits (Casler and Brummer, 2008). Secondly, most of the important forage crops are outbreeding, so variety development is usually based on population improvement via recurrent selection schemes (Posselt, 2010; Conaghan and Casler, 2011). These factors probably contribute to the low genetic gains achieved in forages, but other factors have been suggested, including a lack of a harvest index trait to breed for, inability to exploit heterosis and a large number of target traits with no or negative correlation between them (Casler and Brummer, 2008). Two recent reviews have assessed the prospects for GS in perennial forage crops such as grasses and legumes (Hayes et al., 2013a; Resende et al., 2014). The latter concluded that GS is likely to be most beneficial when phenotypic values of spaced plants do not correlate with those in sward, when within-family selection is difficult or impossible, and for traits that can be assessed only after several years of plot trials. Hayes et al. (2013a) also suggested that significant modifications to most current mass selection breeding schemes in, e.g., perennial ryegrass would be desirable/necessary to implement GS effectively.

However, there is very little empirical data available from forage crops with evaluation of GS performance. Lipka et al. (2014) described the use of GS in predicting breeding values in switchgrass (*Panicum virgatum* L.), a perennial grass which is being developed as an energy crop. They obtained cross validation accuracies of up to 0.52. Slavov et al. (2014) reported prediction accuracies varying between 0.05 (dry matter) and 0.95 (moisture) with an average of 0.57 for 17 traits in the energy grass, *Miscanthus sinensis*. Both used association panels as the training and validation population. Recently, an empirical study of genomic prediction of biomass yield in tetraploid alfalfa reported prediction accuracies between 0.21 and 0.60 depending on the breeding cycle (Li et al., 2015). The authors concluded that the selection efficiencies per unit time based on GS were better than for phenotypic selection. To our knowledge, no empirical data have been published of GS performance in perennial ryegrass, the most important forage crops in temperate grassland agriculture. Here we report our first results of an evaluation of GS in the populations from a long standing and successful recurrent selection breeding program at the Institute of Biological, Environmental, and Rural Sciences (IBERSs). The current populations were established in the late 1980’s from a relatively small founder population, and have now been through up to 14 generations of selection and recombination. We have used current and some historical phenotypic data from plot trials of half sib progeny of mother-plants in combination with genotypic data from the mother-plants. Higher prediction accuracies were obtained for traits related to forage quality, particularly water soluble carbohydrates (WSCs) and digestibility (DMD) than for biomass yield. For most trait-training population combinations the ridge regression BLUP prediction method outperformed the three ML methods employed here. We discuss possible explanations for the results as well as potential ways of improving prediction accuracies particularly for biomass yield.

## Materials and Methods

### Plant Material and Breeding Cycle

**FIGURE 1:**
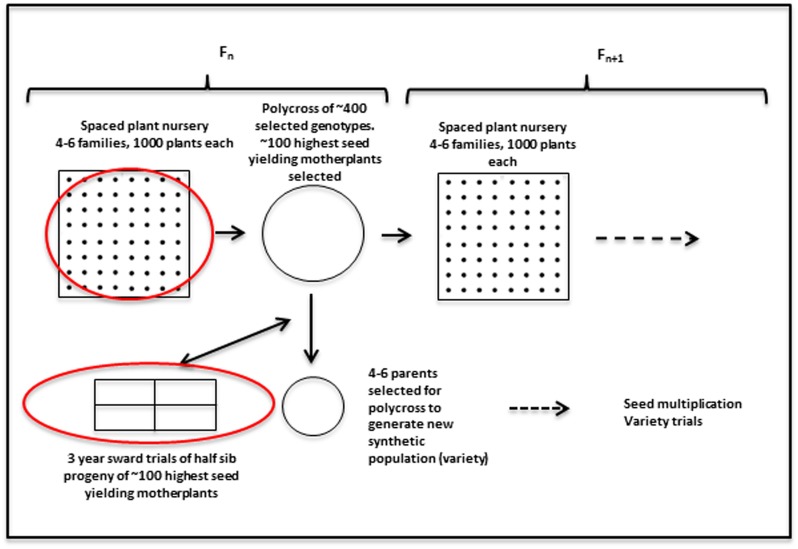
**Outline of the recurrent selection breeding program in *L. perenne*.** The red circles indicate stages where genomic selection (GS) can be implemented, given availability of genotypic, and phenotypic data. The work described here is aimed at utilizing the phenotypic data from the sward trials to facilitate prediction of the best mother-plants for new variety production.

Plant material from the perennial ryegrass breeding populations was used to obtain genotypic and phenotypic data. In order to put the data collection into context, a brief description of the breeding cycle is given. It is also illustrated in **Figure 1.** Any given cycle starts with a polycross of about 400–600 plants from four to six families. Those parents have been collected from spaced plant field plots. Approximately 100 of the highest seed yielding mother-plants are selected to provide half sib progeny for evaluation in sward plot trials. Four replicate plots of the half sib progeny are evaluated over three growing seasons. Biomass yield was recorded for seven cuts each year for the first 2 years, and material from cuts 4 and 5 in the 1st year was used to obtain estimates of dry matter digestibility (DMD), WSCs and nitrogen, with near infrared reflectance spectroscopy (NIRS) (Lister and Dhanoa, 1998). The mean of results from those two cuts were used in the present analysis. At several stages during all three growing seasons persistency was assessed by scoring ground cover visually on a scale of 0–9. In the breeding program the phenotypic data are used to select three–five parents from the mother-plants for poly crossing to obtain a synthetic population for variety trials. The results are also used to inform the selection of 3–6 half-sib families for each new generation. Around 400–600 genotypes from the spaced plant trials of 1000 plants from each family are selected for poly crossing. However, other factors, such as plant stature, disease resistance, and winter survival are also taken into consideration in this selection.

Broad sense heritabilities were calculated as follows:

$$H_{B}^{2}=\frac{\sigma_{G}^{2}}{\sigma_{G}^{2}+\sigma_{E}^{2}}$$

where 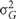 is the genetic variance, and 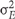 is the residual error variance. The variance components were obtained from a one-way analysis of variance of each of the traits separately. The standard deviation was obtained via leave-one-out Jacknife analysis.

### Genotyping and Linkage Disequilibrium

A 3K Illumina Infinium iSelect Array was used for genotyping of the mother-plants. The SNPs in the array were identified on the basis of polymorphisms in transcriptome libraries from perennial ryegrass plants representing six diverse populations. The development and validation of this array was described in detail previously (Blackmore et al., 2015). The DNA was extracted from leaf material of the mother-plants from each generation as described (Skøt et al., 2011), except for the F12 generation. None of the mother-plants from that generation are in existence, so the DNA was obtained from the husks of the seed derived from the respective mother-plants. In total, DNA samples of sufficient quality were obtained from 86 mother-plants of the F12 generation. After allele calling in the Illumina GenomeStudio software, the genotypic scores were converted to -1, 0, and 1 for input into the various prediction models.

Linkage disequilibrium data (*r^2^*) were obtained using a consensus genetic map containing 1670 markers from the 3 K Infinium Array as described in Blackmore et al. (2015). The LD landscape plots were generated based on an R script described earlier (Wang et al., 2013), but modified and improved for *L. perenne*.

### Training and Test Populations

**FIGURE 2:**
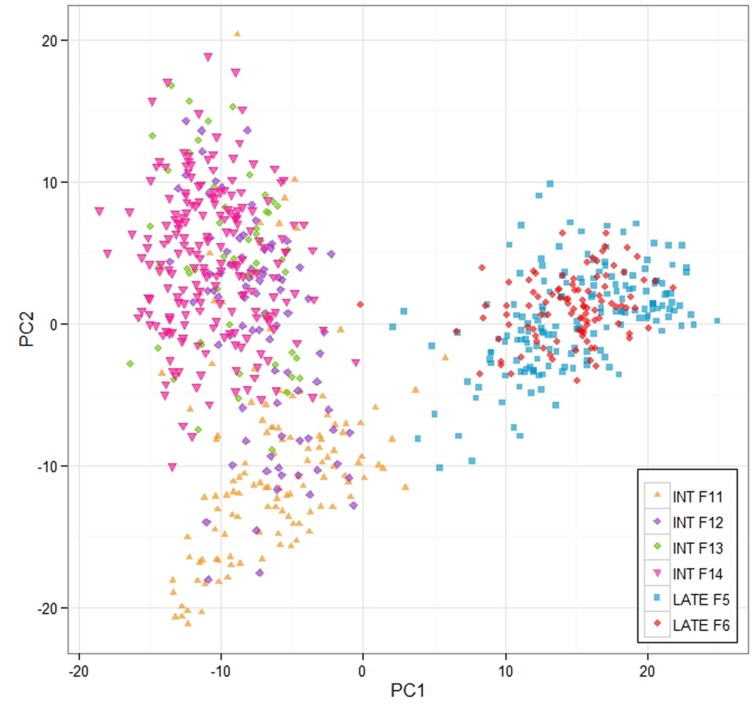
**Principle components analysis of the breeding populations.** The analysis and the plot was based on the 3 K Infinium Array SNP data, and the analysis was implemented in R.

This work was aimed at making genomic predictions of the breeding values of the 100 mother plants of the F14 generation based on training populations consisting of various parts of the previous generations of both the intermediate- and late flowering breeding populations. We wanted to assess the effect of training population size and relatedness to F14 on prediction ability, and also to compare a number of different prediction models in terms of their performance. Three training populations were used. The first was based on the F13 generation, which is closest genetically to F14 (see **Figure 2**). It consisted of 54 mother plants. The second included data from all the intermediate-flowering generations for which we have genotypic and phenotypic data, namely F11, F12, and F13 (this training population is referred to as ‘INT’). The size of that training population was 259. Finally, we also included the late-flowering population F5. This brought the training population size up to 364 (we refer to this training population as ‘ALL’).

All phenotypic data were normalized with respect to each sub-population’s mean and scaled to have variance 1. Thus, hybrid phenotypes F11 + F12 + F13 and F5 + F11 + F12 + F13 do not have variance of exactly 1.

### Prediction Models

We investigated predictive abilities of four methods: GBLUP from statistical genomics and three ML methods. The advantage of GBLUP compared to standard multivariate regression is the ability to cope with the p >> n situation and prevent overfitting via the penalty mechanism. We use GBLUP as the benchmark method against which we compared the three ML models.

We used two tree-based methods: random forests (RF) (Breiman, 2001) and boosted trees GBM (Friedman, 2001). Both methods are non-parametric and make no assumptions about the distribution or any other properties of the data they are applied to, which is an advantage.

For RF we have used the standard values for the number of variables considered at each split (1/3 of the total number), a minimum of five observations per terminal node; trees were grown to their maximal depth and were not pruned and we have grown 500 trees per forest.

For GBM we have used a shrinkage parameter (which discounts each successive tree to avoid overfitting) of 0.01, subsampling rate (proportion of data used to construct each tree) of 0.5 and trees of depth 5, of which have grown 1500 per model.

Thirdly, we used k-nearest neighbors algorithm (KNN) – a model that predicts each new sample point based on the values of its nearest (according to some metric) neighbors in the training set. In KNN regression this prediction is just the average over the values in the neighborhood. This is an example of a *lazy learning* method – generalization beyond training data only occurs when test data is introduced. The advantage of the method is its simplicity and ease of use (one effectively only has one tuning parameter, *k*, the number of neighbors to consider for each new instance) and in the context of GS – the fact that genetic relatedness of plants in the training and test populations is exploited as only plants genetically close to the target are used to calculate each GEBV. For each trait we used the optimal number of neighbors chosen via cross-validation on the corresponding training population (between 1 and 10 for the F13 training population, between 3 and 20 for INT and between 4 and 26 for ALL).

Performance of each model was assessed by calculating Spearman’s rank correlation (*r*(*y, GEBV*) between the corresponding predicted values and the observed F14 phenotypic values.

All analysis was done in R (R Core Development Team, 2014); we used the gbm package for GBM, randomForest for RF, FNN for KNN (Hastie et al., 2009) and rrBLUP (Endelman, 2011) for BLUP.

## Results

### Phenotypic Data and Heritabilities

The phenotypic data were obtained from sward trials derived from half-sib progeny of the 100 or so mother-plants of each generation. The quality traits, such as digestibility, WSCs and nitrogen tended to have higher heritability than the yield-related traits (**Table 1**). There is also variation between years and cuts, highlighting the effects of time. The heritabilities for the biomass yields in the 2nd year tended to be lower than for the 1st year, particularly for F14, but also for the other Intermediate generations.

**Table 1:** Broad-sense heritability for different generations and traits of the breeding populations.

Trait/Population	F5	F11	F12	F13	F14
totaly7c_yr1	0.06 (0.04)	0.22 (0.05)	0.16 (0.06)	0.40 (0.08)	0.19 (0.05)
totaly7c_yr2	0.23 (0.05)	0.02 (0.04)	0.03 (0.04)	0.34 (0.08)	0.08 (0.04)
conscuty_yr1	0.12 (0.05)	0.19 (0.05)	0.16 (0.06)	0.17 (0.06)	0.20 (0.05)
conscuty_yr2	0.27 (0.05)	0.21 (0.05)	0.08 (0.05)	0.17 (0.07)	0.11 (0.05)
vegyld_yr1	0.11 (0.05)	0.46 (0.05)	0.07 (0.05)	0.44 (0.08)	0.18 (0.05)
vegyld_yr2	0.20 (0.06)	0.03 (0.04)	0.05 (0.05)	0.34 (0.07)	0.11 (0.05)
gcscore_yr1	0	0.22 (0.05)	0.30 (0.06)	0.19 (0.06)	0.19 (0.04)
gcscore_yr2	0.27 (0.04)	0.33 (0.05)	0.34 (0.06)	0.12 (0.07)	0.26 (0.05)
dmd	0.17 (0.05)	0.40 (0.05)	0.08 (0.05)	0.59 (0.06)	0.52 (0.05)
n	0.24 (0.04)	0.37 (0.05)	0.03 (0.04)	0.35 (0.08)	0.23 (0.06)
wsc	0.35 (0.06)	0.37 (0.05)	0.22 (0.06)	0.41 (0.08)	0.42 (0.05)


*Standard deviations are in brackets. Trait identification: total7c, total biomass yield over all 7 cuts; conscuty, Yield of conservation cut, i.e., second cut; vegyld, Total biomass yield minus conservation cut; gcscore, Ground cover score; dmd, Dry matter digestibility (%); n, nitrogen (%); wsc, Water soluble carbohydrates (%).*

### Structure of the Breeding Populations

**FIGURE 3:**
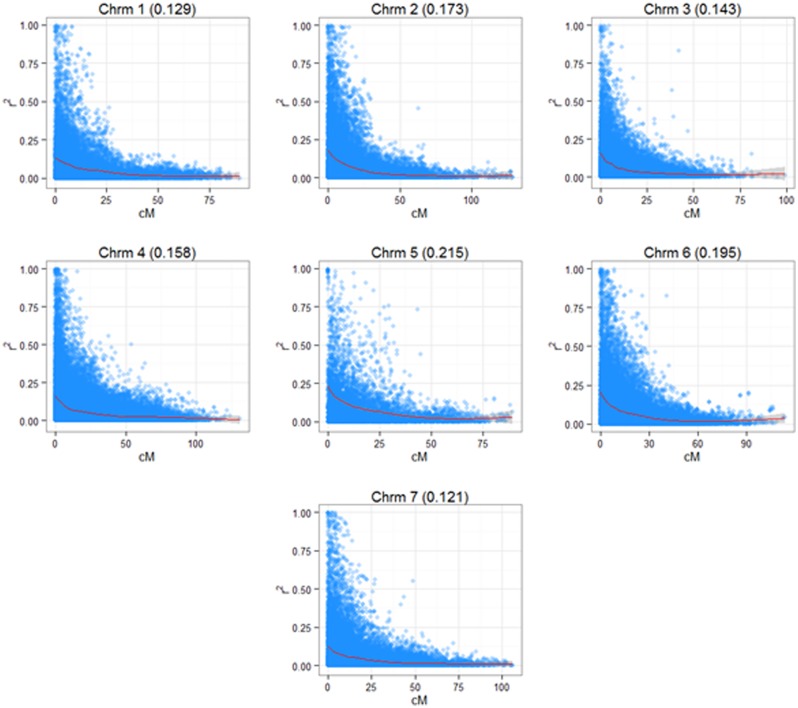
**Linkage disequilibrium (LD) in the seven chromosomes of *L. perenne.*** The diagrams show the pairwise LD (*r^2^*) based on a consensus map derived from three mapping families (see Materials and Methods) with a superimposed cubic smoothing spline.

A 3K SNP Infinium array was used as a platform for genotyping the ryegrass breeding populations (Blackmore et al., 2015). **Figure 2** shows the first two principal components from a PCA analysis on the full genotypic data set, Intermediate F11–14, Late F5–F6 (note that F6 was not used in the analysis elsewhere, since no phenotypic information for it was available at the time of writing, but was included in the PCA analysis, since genotype data were available). The first principle component clearly separates the genotypes in two clusters, one containing the Intermediate population and one containing the Late. The two generations of the Late breeding populations, F5 and F6 form one single cluster, while the Intermediate generations separate along the second principle component. While F13 and F14 form one cluster, F12 and in particular F11 are partially separated from the F13–F14 cluster. LD in the total breeding population is illustrated in two ways. **Figure 3** shows r^2^ between pairs of markers against the corresponding pairwise distances for each of the seven chromosomes. The average distance between consecutive markers is given in brackets above each plot. The average LD for each pairwise marker distance ranged from 0.121 to 0.215. **Supplementary Figure S1** shows landscape and heat-map plots, and they demonstrate that the average pairwise LD ignores some local variations in LD along the chromosomes. The landscape plots and heat-maps show the presence of some hotspots of LD particularly on chromosomes 2 and 6, while the overall level of LD fluctuates between 0.1 and 0.2.

### Genomic Predictions

There are two phases where GS can potentially accelerate the breeding program (**Figure 1**). One is at the spaced plant nursery stage where genotypic information of all the mother plants could assist in the selection of the families being taken forward to the next generation. We do not yet have that information. The other stage is the selection of parents for a new variety or synthetic population, and this is the focus of this first experiment. This is based on genotypic information from the 100 or so mother-plants selected for sward trials of its half sib progeny. We compared four prediction models for the three training sets. The results, recorded as correlations between genomically predicted values and phenotypic data, are summarized in **Tables 2**–**4.** All four methods were poor at predicting the conservation cut yield, while the predictions of total yield were slightly better overall and for vegetative yield even better. For most traits the BLUP method outperformed the other methods (see **Tables 2**–**4**). RF was the second best method with KNN and GBM trailing behind. The highest correlation between observed and predicted values was observed for the forage quality traits, particularly WSCs. This was especially pronounced for the BLUP method, where the correlation approached 0.6 when the INT and INT + F5 = ALL was used as a training population. There was, however, no consistent pattern to the effect of the training population. For BLUP and RF a trend toward better performance was discernible with increasing size of the training population, particularly for the quality traits. However, even that was not entirely consistent. For example DMD had the highest accuracy with F13 as the training population (**Tables 2**–**4**). For the yield based traits, the best prediction accuracies were generally found in the 1st year harvests for the BLUP method (**Tables 2**–**4**). For the two largest training populations (INT and ALL), the prediction accuracy for ground cover (gcscore) was higher in year 2 than in year 1. Data for ground cover in year 3 is not yet available for F14, so prediction accuracies could not be calculated. Of the three biomass yield related traits the highest prediction accuracies were obtained with the BLUP method. The prediction accuracies for these traits were all higher for year 1 data with the BLUP model.

**Table 2:** Correlation between observed phenotyped and GEBV predicted by the four methods trained on F13.

F14/F13	BLUP	KNN	RF	GBM
totaly7c_yr1	0.095	**0.234**	-0.025	0.03
totaly7c_yr2	**0.139**	0.078	0.025	0.059
conscuty_yr1	0.013	0.048	-0.013	**0.145**
conscuty_yr2	-0.009	-0.046	-0.084	0.053
vegyld_yr1	**0.328**	0.188	0.12	0.191
vegyld_yr2	**0.167**	0.103	0.13	0.116
gcscore_yr1	0.345	0.25	**0.392**	0.354
gcscore_yr2	**0.268**	0.143	0.242	0.232
dmd	0.441	0.281	**0.45**	0.188
n	**0.319**	0.104	0.172	0.241
wsc	**0.454**	0.334	0.267	0.303


*The highest prediction accuracy for each trait is highlighted in bold. Trait identification is as described in **Table 1.***

**Table 3:** Correlation between observed phenotyped and GEBV predicted by the four methods trained on INT (Intermediate F11 + F12 + F13).

F14/INT	BLUP	KNN	RF	GBM
totaly7c_yr1	**0.275**	-0.015	0.09	0.223
totaly7c_yr2	-0.013	0.067	0.057	-0.005
conscuty_yr1	**0.114**	0.005	0.14	0.054
conscuty_yr2	-0.048	0.062	-0.105	-0.071
vegyld_yr1	**0.315**	0.093	0.295	0.134
vegyld_yr2	0.044	0.055	**0.071**	0.01
gcscore_yr1	**0.28**	-0.038	0.124	-0.206
gcscore_yr2	**0.339**	0.168	0.266	0.182
dmd	**0.396**	0.148	0.347	0.132
n	0.290	0.166	**0.357**	0.041
wsc	**0.590**	0.353	0.365	0.292


*The highest prediction accuracy for each trait is highlighted in bold.*

**Table 4:** Correlation between observed phenotyped and GEBV predicted by the four methods trained on ALL (INT + Late F5).

F14/ALL	BLUP	KNN	RF	GBM
totaly7c_yr1	**0.224**	-0.066	0.135	0.012
totaly7c_yr2	**0.082**	-0.026	0.044	-0.087
conscuty_yr1	**0.078**	0.041	0.012	0.048
conscuty_yr2	0.001	-0.016	-0.036	**0.067**
vegyld_yr1	**0.281**	0.157	0.264	0.109
vegyld_yr2	0.095	0.078	**0.155**	0.064
gcscore_yr1	**0.234**	-0.005	0.01	-0.205
gcscore_yr2	**0.369**	0.136	0.252	0.175
dmd	**0.414**	0.239	0.353	0.076
n	0.314	**0.337**	0.314	0.231
wsc	**0.598**	0.366	0.402	0.277


*The highest prediction accuracy for each trait is highlighted in bold.*

## Discussion

### Accuracy and Prediction Model

This work represents the first empirical evaluation of GS in perennial ryegrass, the most important temperate forage grass crop. We tested four prediction models and three training populations in order to assess the effect of the method and the size and composition of the training populations. The comparison between the different prediction models was most straightforward, since this can be done for each training population. Overall BLUP was the best performing method but ML techniques were reasonably successful on the F13 training population, where they outperformed BLUP for 4 out of 11 traits (**Table 2**). Traits with higher heritability consistently gave better prediction accuracy. This was particularly evident for DMD and WSCs (**Tables 2**–**4**), which both have the highest heritability (**Table 1**) and the highest prediction accuracy. One of the characteristics of the quality traits is that the frequency distribution in terms of percentage of dry matter was unimodal even after combining the data for different generations, while yield-related traits differ markedly between years, location and generation, and so have bi- or tri-modal frequency distributions. We have tried to mitigate these environmental effects here by scaling the trait values separately for each generation, and also normalizing them against phenotypic values of control varieties. Furthermore, we considered yield-related traits in different years as different traits. The effectiveness of this is very much dependent on the presence or absence of genotype by environment interaction (G × E). If there is considerable G × E the predictions will be different for different years. Furthermore, variation in heritability between years is also likely to have an effect on accuracy. **Tables 2**–**4** show that there are differences in prediction accuracies between years, and in particular differences between the effects of composition of the training population. The generally lower prediction accuracies for the yield-related traits are consistent with their lower heritability (**Table 1**).

### Accuracy and Training Population

The relationship between size and composition of training population on the one hand and prediction accuracy on the other was complex, and more difficult to interpret. This is because the change in training population size is compounded by the population structure (**Figure 2**). For 16 of the 44 trait/prediction method combinations, the prediction accuracies increased when replacing F13 with all intermediate generations, i.e., F11 + F12 + F13. This increased the training population size from 54 to 259, so everything else being equal, an increase in accuracy would be expected. However, for more than a half of the combinations this was not the case. A further increase in the training population with 105 individuals from the Late F5 generation did not improve accuracy appreciably for most of the traits/methods combinations. Population structure could partially explain this result. While the most obvious difference was between the Intermediate and the Late groups, F12 and particularly F11 diverged from the F13/F14 cluster (**Figure 2**). The genetic distance between the generations could possibly explain why we do not see a consistent increase in prediction accuracy with an increase in training population size. This may be equivalent to the situation in animal breeding where there are examples of loss in predication accuracy across breed predictions as compared to within breed (Daetwyler et al., 2012; Erbe et al., 2012). Due to the limited extent of LD across breeds, it is estimated that large cross-breed reference populations are needed (Goddard and Hayes, 2009). In the ryegrass breeding populations, the genetic separation is most likely driven by a combination of deliberate selection and genetic drift, the latter of which is more important in a population with a small effective population size. A small effective population size limits the number of genes causing an effect on a trait. The original number of founders of the Intermediate population was low (10), but polycrossing in subsequent generations included approximately 400 plants, and thus helped generate a great many more haplotypes than the original 20. A combination of a larger effective population size and genetic separation requires a higher coverage of SNP markers. An estimate of the effective population size of the breeding population can be obtained as described from the empirical estimates of LD we have obtained. The expectation of LD is given by *r^2^ =* 1/(4N_e_c + 1), where N_e_ is the effective population size, and c is the distance between adjacent marker in Morgans (Sved, 1971). Assuming an LD estimate of 0.1 (**Figure 3**, **Supplementary Figure S1**) and an average distance of 0.003 Morgans between adjacent markers, the effective population size is 281. This is somewhere between the original number of founders (10) and the number of parents in the polycrosses of selected spaced plants at each generation (400). Given a prediction accuracy of *r =* ∼0.5 and heritability of 0.4 (approximate values for WSC), one would expect to require a training population size of 2983 unrelated individuals. The appropriate values have been substituted in the following equation: *r^2^ = Nh^2^/(Nh^2^ + M_e_)*, where *M_e_ = 2N_e_L/ln(4N_e_L). L* is the genome size in Morgans (eight for *L. perenne*), *h^2^* is heritability, and *N* is the size of the training population (Meuwissen, 2009). The prediction accuracies we have obtained here, at least for the quality traits, with a much smaller training population is likely due to the strong relatedness of the training population to the test population. Relatedness would thus appear to be a very important factor determining the success of GS.

### Genomic Prediction in Future Ryegrass Breeding

The breeding program described here is similar to the suggested generalized scheme for implementation of GS in forage crops (Hayes et al., 2013a). It thus represents a suitable template for this initial evaluation of prediction accuracies. The particular methodology of the ryegrass breeding program, however, presents a challenge. The need to use sward trials to obtain realistic phenotypic data, especially for biomass-related traits, means that the prediction accuracies in our implementation are based on genotypic data from mother-plants and phenotypic data from sward derived from seed of half-sib progeny of the mother-plants. Given the mixture of genotypes in such a sward this is likely to lower the obtainable prediction accuracies. If genotypic data were available from all the potential pollen donors in the poly crosses, it would enable us to predict allele frequencies in the progeny, but this was not economically feasible. In a white spruce population it was also found that prediction accuracies decreased markedly when the validation population was unrelated (or had unknown relationship) to the training population (Beaulieu et al., 2014). Prediction accuracies between 0.327 and 0.435 were found where the relationship between training and validation population was closest. The larger training population and number of markers (1694 and 6358, respectively) could explain the more consistent results across traits compared to our results. Nevertheless, the prediction accuracies for the forage quality traits are comparable to those in white spruce. In switchgrass prediction accuracies for a range of morphological and quality traits varied between 0 and 0.55, and are thus also within the same range as ryegrass. In alfalfa it was recently reported that genomic prediction accuracies of biomass yield were highest within the same breeding cycle compared to prediction across cycles (Li et al., 2015). This is consistent with the situation in the ryegrass breeding program. The higher and more consistent accuracies reported in alfalfa is most likely due to higher heritabilities for the biomass traits, and that the phenotypic and genotypic data were obtained from the same spaced plants, and not half sib progeny.

As has been pointed out previously (Daetwyler et al., 2012; Liu et al., 2015) prediction accuracies are determined to a large extent by genomic relationships (population structure) and LD. Given the limited number of markers used in this study and the extent of LD in the breeding populations, it would seem likely that the accuracies obtained here are attributable to the capture of the relatedness between genotypes. In other words, the closer the relationship between training population and test population, the fewer markers are required to obtain a given accuracy (Liu et al., 2015).

Other factors that influence the accuracy are the environmental factors affecting plants grown in different years. This is highlighted by the variable prediction accuracies between years for the yield related traits (**Tables 2**–**4**). These factors make combining populations into homogenous training sets a non-trivial, and often difficult, task. This also makes tuning hyperparameters of ML models on the training set difficult; for instance, often parameters deemed optimal by tuning on (any of the three) training populations were suboptimal choices when tested on the F14 population. This significantly reduced accuracy results produced by the three, usually very powerful, ML models on the F14 test set. Another reason for the comparatively good performance of GBLUP is the fact that biomass-related and forage quality traits are controlled by many QTLs with small effects, a situation which is optimal for GBLUP. However, for some of the combinations RF performed better than GBLUP (e.g., **Table 2**, DMD). If a ML prediction method was consistently outperforming other methods, it would be easy to “mix and match” prediction methods to traits. At present the results are not sufficiently consistent to consider this. Obtaining more biomass yield data from different sites (environments) should improve prediction accuracies.

In this work we considered the phenotypic performance in sward, and the GEBV values obtained from this can be used to inform which parents to select for generating a potential new synthetic population or variety (**Figure 1**). For this purpose prediction accuracies would need to be as high as the predictions based on phenotypic evaluation. While this is not the case, the GEBVs can also be used to assist in the selection of families (seed of a mother plant) to select for the next generation of the spaced plant nursery. The long running IBERS breeding scheme outlined in **Figure 1** is in fact very similar to the one proposed in a recent review (Hayes et al., 2013a). As we obtain more and more complete information of the pedigree of the breeding populations from the genotypic data, we can begin to make informed decisions to maximize the genetic variation in the breeding population, and perhaps even reduce the size of it, while maintaining variation. The improvement of GEBVs over generations will eventually lead to a situation where they can compete with the phenotypic evaluation, and thus begin to save time (Hayes et al., 2013a).

We demonstrated the use of a GS approach, in which one standard statistical method and three ML methods were compared for predicting GEBVs in *L. perenne*. The results are most encouraging for forage quality traits, such as WSCs and DMD, and highlight several important points. Improved prediction accuracies are desirable for the yield related traits, particularly in the second year. A larger training population closely related to the validation population and a larger number of markers would probably improve accuracy. However, low heritability of a trait makes such improvements more difficult to achieve. Future work might involve devising more efficient ways of combining different sub-populations, since small training population size together with genome wide LD (**Figure 3**, **Supplementary Figure S1**) limit the prediction ability in GS. It would also be very interesting to incorporate meteorological data into ML models thus not only accounting for some of the environmental effects, but also uncovering G × E interactions.

## Author Contributions

NG conceived the work, analyzed the data and wrote the paper, AL provided phenotypic data from the breeding programme and analysed some of the data, MH conceived the work, and developed the SNP CHIP, Andi Lovatt maintained and propagated the plants and provided technical assistance, KPS ran the SNP CHIP analysis, RK provided technical assistance with DNA extraction and marker analysis, TB developed the SNP CHIP and provided the marker data, DT developed the genetic map used as the basis for the LD analyses, RDK conceived the work and supervised the data analysis, IA conceived the work, WP conceived the work, LS conceived and supervised the work, analyzed some of the data and wrote the paper.

## Conflict of Interest Statement

The authors declare that the research was conducted in the absence of any commercial or financial relationships that could be construed as a potential conflict of interest.
