# Supplementary material for: Implementation of Genomic Prediction in Lolium perenne (L.) Breeding Populations
Source: Front Plant Sci. 2016 Feb 12;7:133. doi: 10.3389/fpls.2016.00133 (PMC4751346; doi:10.3389/fpls.2016.00133)
Supplement: Supplementary file 1 [file Data_Sheet_1.PDF]

Supplementary Material

Implementation of genomic prediction in *Lolium perenne* (L.) breeding populations

Nastasiya F. Grinberg[1], Alan Lovatt[2], Matt Hegarty[2], Andi Lovatt[2], Kirsten P. Skøt[2], Rhys Kelly[2], Tina Blackmore[2], Danny Thorogood[2], Ross King[1], Ian Armstead[2], Wayne Powell[2,3], Leif Skøt[2*]

[1]Manchester Institute of Biotechnology, University of Manchester, Manchester, UK

[2]Institute of Biological, Environmental and Rural Sciences, Aberystwyth University, Aberystwyth, UK

[3]CGIAR Consortium, CGIAR Consortium Office, 1000 Avenue Agropolis 34394, Montpellier, France

*** Correspondence:** Leif Skøt, E-mail: lfs@aber.ac.uk

1.1 Supplementary Figures

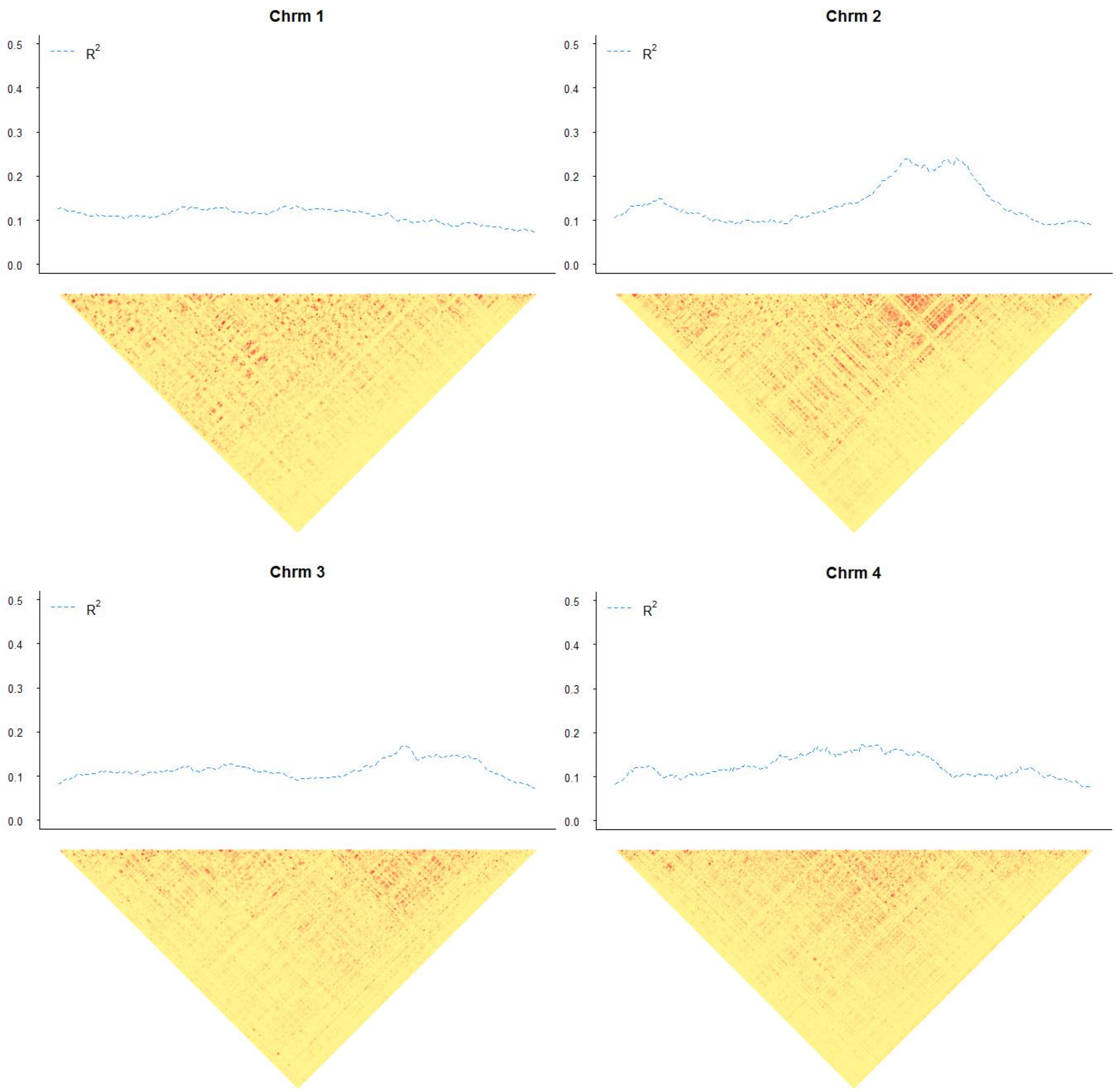

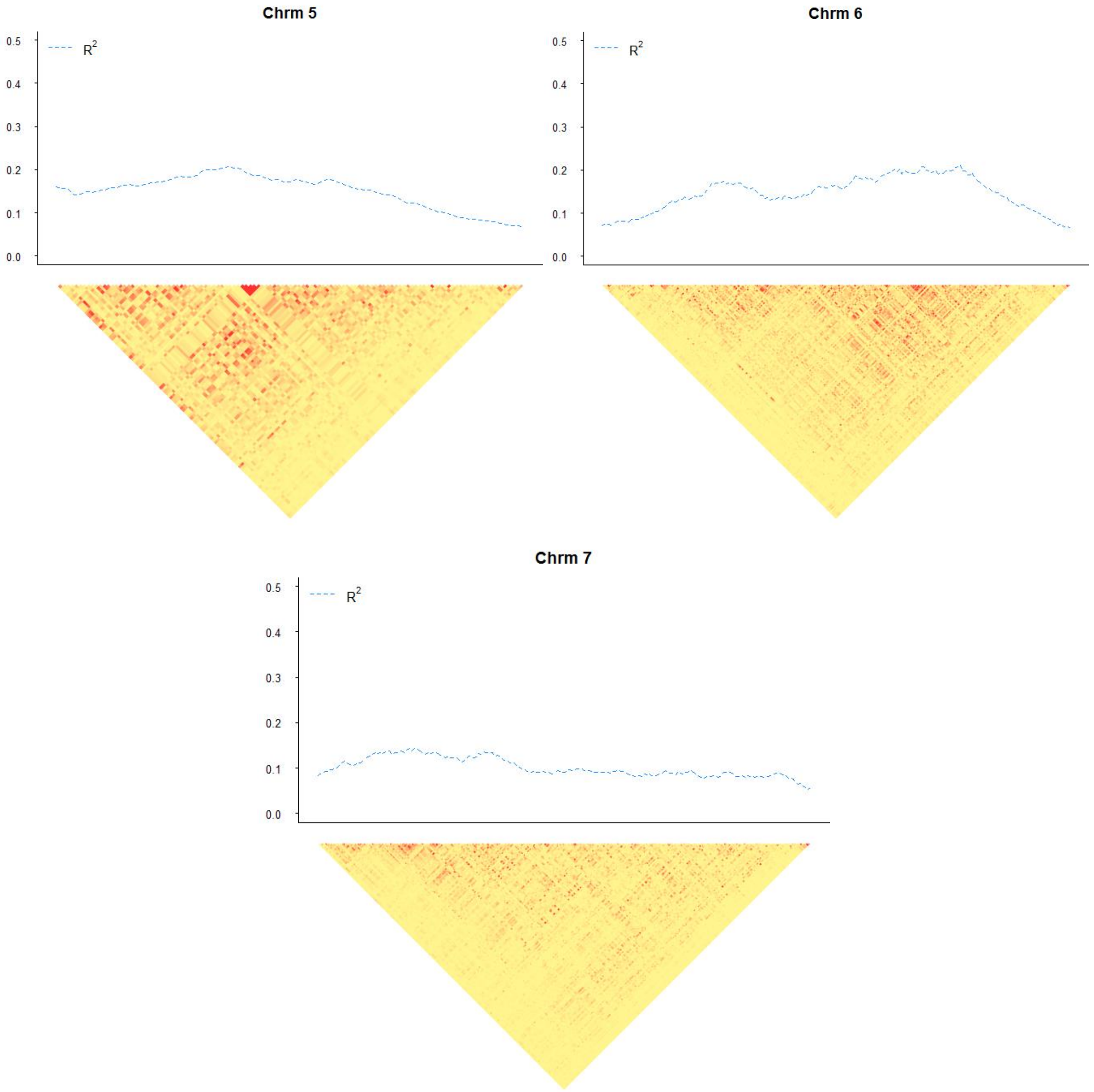

Supplementary Figure 1. Landscape plot and heatmaps of linkage disequilibrium in the *L. perenne* breeding populations. The landscape plots were based on a windows size of n=40, as described in Materials and Methods.
